# Predictive Effect of FT3 Within the Euthyroid Range on HDL‐C in Patients With Type 2 Diabetes: A Cross‐Sectional Analysis of Inpatients in China

**DOI:** 10.1002/jcla.70029

**Published:** 2025-03-28

**Authors:** Jinmei Xu, Shangshuang Zhao, Yan Wang, Jun Han

**Affiliations:** ^1^ Department of Endocrinology and Metabolism The Fourth Affiliated Hospital, Harbin Medical University Harbin China

**Keywords:** free triiodothyronine, high‐density lipoprotein cholesterol, type 2 diabetes

## Abstract

**Background:**

Evidence for assessing the relationship between free triiodothyronine (FT3) and high‐density lipoprotein cholesterol (HDL‐C) remains limited. Therefore, the purpose of our study is to evaluate the relationship between FT3 and HDL‐C in patients with type 2 diabetes.

**Methods:**

From June 2022 to October 2023, 3011 patients with normal thyroid function and diagnosed with type 2 diabetes mellitus (T2DM) were collected continuously and non‐selectively in a Chinese hospital. Then, we used a logistic regression model to explore the relationship between FT3 and HDL‐C. Smooth curve fitting is used to identify the nonlinear relationship between FT3 and HDL‐C.

**Results:**

After adjusting for the influence of relevant factors, FT3 and HDL‐C were negatively correlated −0.02 (−0.04, −0.00; *p* = 0.0162). There is also a nonlinear relationship between FT3 and HDL‐C, with an inflection point of 3.48 pmol/L for FT3 (P for log‐ likelihood ratio test = 0.044).

**Conclusion:**

This study shows that there is a negative correlation and nonlinear relationship between FT3 and HDL‐C in the Chinese population with diabetes. When FT3 is between 2.76–3.48 pmol/L, HDL‐C tends to a stable state; When FT3 is between 3.48–6.45 pmol/L, HDL‐C decreases with the increase of FT3 concentration (According to the reference range used by our hospital, the normal value of serum FT3 is 2.76–6.45 pmol/L). These findings suggest that maintaining FT3 within the range of 2.76 to 3.48 pmol/L may be most beneficial for mitigating the progression of cardiovascular disease in patients with T2DM.

## Graphical abstract

Our research is important for identifying the optimal FT3 range to delay the progression of cardiovascular disease in patients with T2DM. These findings provide valuable insights to guide clinicians in preventing and managing cardiovascular disease in this population.

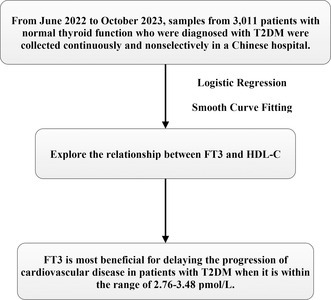

## 1 Introduction

Thyroid hormones are considered catabolic hormones that regulate various metabolic processes, including the synthesis, mobilization, and breakdown of lipids. Hypothyroidism has been reported to be associated with dyslipidemia and an increased risk of atherosclerotic cardiovascular disease [1, 2]. Interestingly, there is now a view that the effects of hypothyroidism on susceptibility to atherosclerosis may extend into the euthyroid range. Several studies have reported an association between higher thyroid‐stimulating hormone (TSH) and lower thyroid hormone levels (still within the normal range) and lipid profiles in euthyroid subjects [3, 4].

Diabetes, especially type 2 diabetes mellitus (T2DM), is primarily associated with lipid abnormalities [5] and is also known to significantly increase the risk of cardiovascular disease [6]. Of note, thyroid dysfunction is more common in patients with diabetes than in the general population [7]. Furthermore, the association between thyroid hormone levels and cardiovascular disease risk factors appears to be amplified by the degree of insulin resistance [8], which may be particularly relevant for T2DM. Therefore, glucose, lipids, and thyroid hormones appear to interact more complexly than previously expected. Recently, several studies have reported that even thyroid function that is relatively low but still within the normal range is more common and may be more dangerous in patients with diabetes [9, 10, 11]. However, there is little research on these issues. Moreover, in most of these studies, the correlation between serum free triiodothyronine (FT3) and high‐density lipoprotein cholesterol (HDL‐C) in patients with T2DM has been rarely clarified.

Taking the above factors into consideration, we investigated the potential association between serum FT3 and HDL‐C in a group of patients with T2DM and normal thyroid function. We hope that the information from this study can help people better understand the relationship between thyroid parameters and lipid profiles and provide data support for the clinical prevention and treatment of T2DM.

## 2 Materials and Methods

### 2.1 Research Subjects

To minimise selection bias, data from all patients diagnosed with T2DM in the Endocrinology Department of the Fourth Affiliated Hospital of Harbin Medical University between June 2022 and October 2023 were collected non‐selectively and consecutively. The hospital's electronic medical record system was updated in June 2022, resulting in the loss of some earlier patient records. To ensure privacy, all patient identity information was encoded using non‐traceable codes. Data were retrieved from the hospital's electronic medical records. This study was approved by the Ethics Committee of the Fourth Affiliated Hospital of Harbin Medical University. A total of 4752 patients were initially included. After applying exclusion criteria, 3011 patients were retained for analysis (average age 56.92 ± 12.56 years), including 1430 males (average age 53.91 ± 12.78 years) and 1581 females (average age 59.65 ± 11.72 years).

**FIGURE 1:**
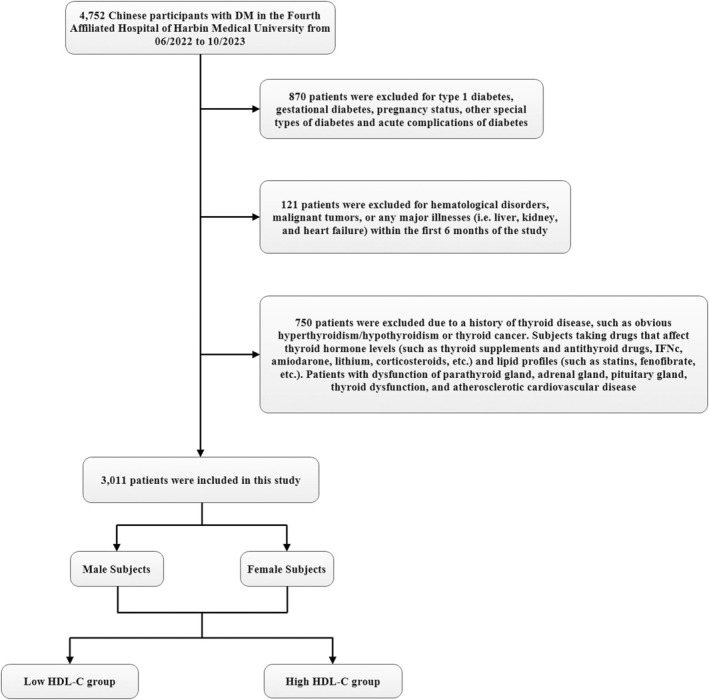
Flowchart of study participants. This figure showed the inclusion of participants. A total of 4752 participants were assessed for eligibility in the original study. The final analysis included 3011 subjects in the present study.

The thyroid function of these patients was determined to be normal based on the reference ranges used at the Harbin Medical University Affiliated Fourth Hospital: serum FT3 2.76–6.45 pmol/L, free thyroxine (FT4) 11.20–23.81 pmol/L, and TSH 0.35–5.10 mIU/L. All subjects included in this study were diagnosed with diabetes according to the diagnostic criteria established by the World Health Organization (WHO) in 1999 [12]. Criteria included: (1) the presence of typical symptoms of diabetes and a random blood glucose level ≥ 11.1 mmol/L; (2) fasting blood glucose ≥ 7.0 mmol/L; (3) a blood glucose level ≥ 11.1 mmol/L 2 h after ingesting 75 g of glucose in an oral glucose tolerance test. For patients without typical diabetes symptoms (such as polydipsia, polyphagia, polyuria, and weight loss), repeat testing was required on another day to confirm the diagnosis. Exclusion criteria: (1) patients with type 1 diabetes, gestational diabetes, pregnancy status, and other special types of diabetes; (2) patients with acute complications of diabetes; (3) patients with blood system diseases, malignant tumors, and any major diseases within 6 months before the study (i.e., liver, kidney, and heart failure); (4) patients with atherosclerotic cardiovascular disease; (5) subjects with a history of thyroid disease, such as overt hyperthyroidism/hypothyroidism, thyroid cancer. Subjects who take drugs that affect thyroid hormone levels (such as thyroid supplements and antithyroid drugs, IFNc, amiodarone, lithium, corticosteroids) and lipid profiles (such as statins, fenofibrate); (6) Patients with parathyroid, adrenal, pituitary dysfunction and abnormal thyroid function. According to the “Guidelines for the Prevention and Treatment of Type 2 Diabetes in China” [13], HDL‐C should be controlled at > 1.0 mmol/L in male patients with T2DM; and should be controlled at > 1.3 mmol/L in female patients with T2DM. In this study, a total of 904 male T2DM patients up to the HDL‐C target; and 400 female T2DM patients up to the HDL‐C target. In different gender groups, we divided them into two groups based on whether HDL‐C up to the standard: Low HDL‐C group and High HDL‐C group (Figure 1) (The fasting blood glucose (FBG) and HDL‐C wer determined to be normal based on the reference ranges used at the Harbin Medical University Affiliated Fourth Hospital: serum FBG 3.89–6.11 mmol/L, HDL‐C 1.04–1.55 mmol/L).

### 2.2 General Conditions and Laboratory Biochemical Indices

The sex, age, height, weight, systolic blood pressure (SBP), diastolic blood pressure (DBP), and diabetic duration of 3011 patients were analyzed retrospectively, and the situation of diabetes was recorded in detail. Body Mass Index (BMI) is calculated using the following formula: BMI (kg/m^2^) = weight (kg) / body height^2^ (m^2^).

All subjects were required to fast for more than 8 h, venous blood was drawn and urine samples were collected the next morning. The biochemical parameters measured included glycosylated hemoglobin (HbA1c) (high performance liquid chromatography method), FBG (hexokinase method), triglyceride (TG) (glucose oxidase (GOD)‐peroxidase (POD) method), total cholesterol (TC) (enzymatic method), HDL‐C (selective inhibition method), low‐density lipoprotein cholesterol (LDL‐C) (peroxidase clearance method), serum creatinine (sCr) (enzymatic method), FT4 (ChemiLuminescence method), FT3 (ChemiLuminescence method), TSH (ChemiLuminescence method), Alanine aminotransferase (ALT) (lactate dehydrogenase method) and aspartate aminotransferase (AST) (malate dehydrogenase (MDH) method). Urinary microalbuminuria, urinary creatinine, and the ratio of urinary microalbuminuria to urinary creatinine (UACR) were determined by immunoturbidimetry (Thyroid hormones promote liver cell synthesis and secretion of transaminase, leading to an increase in ALT and AST levels. The liver is the core organ of human lipid metabolism, responsible for the synthesis, transformation, and excretion of lipids such as cholesterol and triglycerides [14]. Therefore, in exploring the correlation between FT3 and HDL‐C, this study took into account the effects of ALT and AST and included them). These patients were determined to be normal based on the reference ranges used at the Harbin Medical University Affiliated Fourth Hospital: HbA1c 4.0%–6.0%, FBG 3.89–6.11 mmol/L, TG 0.56–1.70 mmol/L, TC < 5.18 mmol/L, HDL‐C 1.04–1.55 mmol/L, LDL‐C < 3.37 mmol/L, sCr 57.0–97.0 μmol/L, ALT 9–50 U/L, AST 15–40 U/L.

### 2.3 Statistical Analysis

All the analyses were performed with the statistical software packages R (The R Foundation) 2 and EmpowerStats3 (X&Y Solutions Inc., Boston, MA). *P*‐values less than 0.05 (two‐sided) were considered statistically significant.

The minor absolute shrinkage and selection operator (LASSO) method is suitable for reducing high‐dimensional data [15, 16] and was used to select the most useful predictive candidates from the dataset. Candidates with non‐zero coefficients are chosen to establish the LASSO model [17].

## 3 Results

### 3.1 Comparison of General Characteristics and Biochemical Indexes Among the Two Groups

The clinical characteristics of male and female subjects are shown in Table 1. Of the 1430 male T2DM patients, 526 (36.78%) patients' HDL‐C ≤ 1.0 mmol/L. Compared with the male Low HDL‐C group, the High HDL‐C group had lower levels of HbA1c and FBG (*p* < 0.01). There were significant differences in the levels of Age, BMI, FT3, FT4, sCr, AST, ALT, TC, TG, and LDL‐C between the two groups (*p* < 0.05). Among 1581 female T2DM patients, 1181 (74.70%) patients' HDL‐C ≤ 1.3 mmol/L. There were significant differences in Age, BMI, SBP, sCr, TC, TG, and LDL‐C levels between the two groups (*p* < 0.05) (Table 1). Next, we will focus on analyzing the correlation between FT3 and HDL‐C. The distribution of FT3 levels among all subjects follows a normal distribution, ranging from 2.76 to 6.31 pmol per liter, with an average FT3 of 4.35 ± 0.56 pmol/L.

**TABLE 1:** Clinical characteristics of the study subjects according to gender.

Variables	Male subjects				Female subjects			
	Low HDL‐C group (*n* = 526)	High HDL‐C group (*n* = 904)	Standardize diff.	*p*	Low HDL‐C group (*n* = 1181)	High HDL‐C group (*n* = 400)	Standardize diff.	*p*
Age (years)	52.03 ± 12.82	55.00 ± 12.63	0.23 (0.13, 0.34)	< 0.001	59.02 ± 12.18	61.50 ± 10.03	0.22 (0.11, 0.34)	< 0.001
Height (m)	1.73 ± 0.06	1.72 ± 0.05	0.22 (0.11, 0.33)	< 0.001	1.61 ± 0.05	1.61 ± 0.05	0.03 (−0.09, 0.14)	0.624
Weight (Kg)	77.44 ± 11.65	75.03 ± 11.77	0.21 (0.10, 0.31)	< 0.001	63.76 ± 9.23	61.83 ± 9.11	0.21 (0.10, 0.32)	< 0.001
BMI (kg/m^2^)	25.76 ± 3.25	25.32 ± 3.38	0.13 (0.03, 0.24)	0.016	24.58 ± 2.99	23.89 ± 3.02	0.23 (0.12, 0.34)	< 0.001
Diabetic duration (years)	4.00 (1.00–10.00)	6.00 (1.00–10.00)	0.10 (−0.01, 0.21)	0.067	7.00 (1.00–13.00)	8.00 (2.00–14.25)	0.07 (−0.04, 0.19)	0.201
SBP (mmHg)	137.33 ± 19.84	138.34 ± 20.00	0.05 (−0.06, 0.16)	0.359	137.06 ± 19.15	139.87 ± 20.40	0.14 (0.03, 0.26)	0.013
DBP (mmHg)	88.12 ± 13.10	86.84 ± 12.44	0.10 (−0.01, 0.21)	0.065	85.26 ± 11.99	84.44 ± 12.06	0.07 (−0.05, 0.18)	0.240
FT3 (pmol/L)	4.44 ± 0.58	4.37 ± 0.58	0.13 (0.02, 0.24)	0.017	4.33 ± 0.55	4.27 ± 0.54	0.11 (−0.00, 0.22)	0.056
FT4 (pmol/L)	18.07 ± 2.40	17.60 ± 2.47	0.19 (0.09, 0.30)	< 0.001	17.64 ± 2.47	17.77 ± 2.43	0.05 (−0.06, 0.17)	0.355
TSH (mIU/L)	2.23 ± 1.05	2.23 ± 1.09	0.00 (−0.10, 0.11)	0.942	2.27 ± 1.06	2.16 ± 1.02	0.11 (−0.01, 0.22)	0.070
HbA1c (%)	8.96 ± 1.92	8.65 ± 2.16	0.15 (0.04, 0.26)	0.007	8.73 ± 2.03	8.52 ± 2.18	0.10 (−0.01, 0.21)	0.083
sCr (mmol/L)	71.80 ± 29.39	66.16 ± 23.87	0.21 (0.10, 0.32)	< 0.001	67.79 ± 27.12	62.02 ± 29.50	0.20 (0.09, 0.32)	< 0.001
AST (U/L)	22.00 (18.00–32.00)	22.00 (18.00–27.00)	0.18 (0.07, 0.29)	< 0.001	21.00 (17.00–28.00)	21.00 (17.00–26.00)	0.03 (−0.08, 0.14)	0.612
ALT (U/L)	25.00 (18.00–39.00)	22.00 (16.00–31.00)	0.25 (0.14, 0.36)	< 0.001	22.00 (16.00–32.00)	19.00 (15.00–26.00)	0.09 (−0.02, 0.21)	0.095
TC (mmol/L)	5.09 ± 1.53	5.45 ± 1.23	0.26 (0.15, 0.37)	< 0.001	5.20 ± 1.35	5.83 ± 1.33	0.47 (0.36, 0.58)	< 0.001
TG (mmol/L)	2.68 (1.66–4.54)	1.56 (1.10–2.25)	0.64 (0.53, 0.75)	< 0.001	1.79 (1.27–2.89)	1.21 (0.92–1.67)	0.55 (0.43, 0.66)	< 0.001
HDL‐C (mmol/L)	0.86 ± 0.10	1.26 ± 0.21	2.38 (2.24, 2.52)	< 0.001	1.04 ± 0.16	1.53 ± 0.20	2.68 (2.54, 2.83)	< 0.001
LDL‐C (mmol/L)	2.51 ± 0.90	3.14 ± 0.97	0.67 (0.56, 0.78)	< 0.001	2.89 ± 0.97	3.32 ± 1.10	0.42 (0.30, 0.53)	< 0.001
FBG (mmol/L)	9.94 ± 4.04	9.27 ± 4.26	0.16 (0.05, 0.27)	0.004	9.35 ± 3.86	9.02 ± 3.97	0.09 (−0.03, 0.20)	0.137
UACR (mg/g)	22.35 (9.66–86.35)	18.35 (8.37–64.12)	0.04 (−0.07, 0.15)	0.470	18.80 (8.53–65.10)	16.30 (7.76–41.33)	0.01 (−0.10, 0.12)	0.838

*Note:* Results in the table: Median (Q1−Q3)/N(%). *P* value*: if it is a continuous variable, it can be obtained by KruskalWallis rank sum test. If the theoretical number of counting variable is less than 10, it can be obtained by Fisher exact probability test.

Abbreviations: ALT, alanine aminotransferase; AST, aspartate transaminase; BMI, body mass index; DBP, diastolic blood pressure; eGFR, glomerular filtration rates; FBG, fasting blood glucose; FT3, free triiodothyronine; FT4, free thyroxine; HbA1c, glycosylated hemoglobin; HDL‐C, high‐density lipoprotein cholesterol; LDL‐C, low‐density lipoprotein cholesterol; SBP, systolic blood pressure; sCr, serum creatinine; TC, total cholesterol; TG, triglycerides; TSH, thyroid‐stimulating hormone; UACR, urinary‐albuminuria‐creatinine ratio.

### 3.2 LASSO Regression in Dataset

**FIGURE 2:**
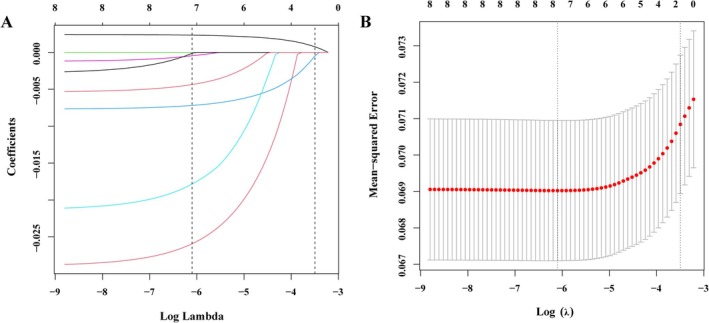
Demographic and clinical feature selection using the LASSO binary logistic regression model. (A) Optimal candidate (Lambda) selection in the LASSO model used 10‐fold cross‐validation via minimum criteria. The area under the receiver operating characteristic curve was plotted versus log (Lambda). Dotted vertical lines were drawn at the optimal values by using the minimum criteria and the 1 SE of the minimum standards. (B) LASSO coefficient profiles of the 17 candidates. A coefficient profile plot was produced against the log (Lambda) sequence. A vertical line was drawn at the value selected using 10‐fold cross‐validation, where optimal Lambda resulted in 14 candidates with non‐zero coefficients (Lambda = 0.0022).

In the data set, 17 risk factors of patient demographic, clinical, and laboratory indicators were included in LASSO regression analysis (Figure 2). Variables with non‐zero coefficients in the LASSO regression model are considered to be related to HDL‐C. They were selected for further studies, including Age, Sex, BMI, FT3, FT4, TSH, and HbA1c. The Lambda value has the minimum average cross‐validation error of 0.0022.

### 3.3 Smooth Curve Fitting and Threshold Effect Analysis

**FIGURE 3:**
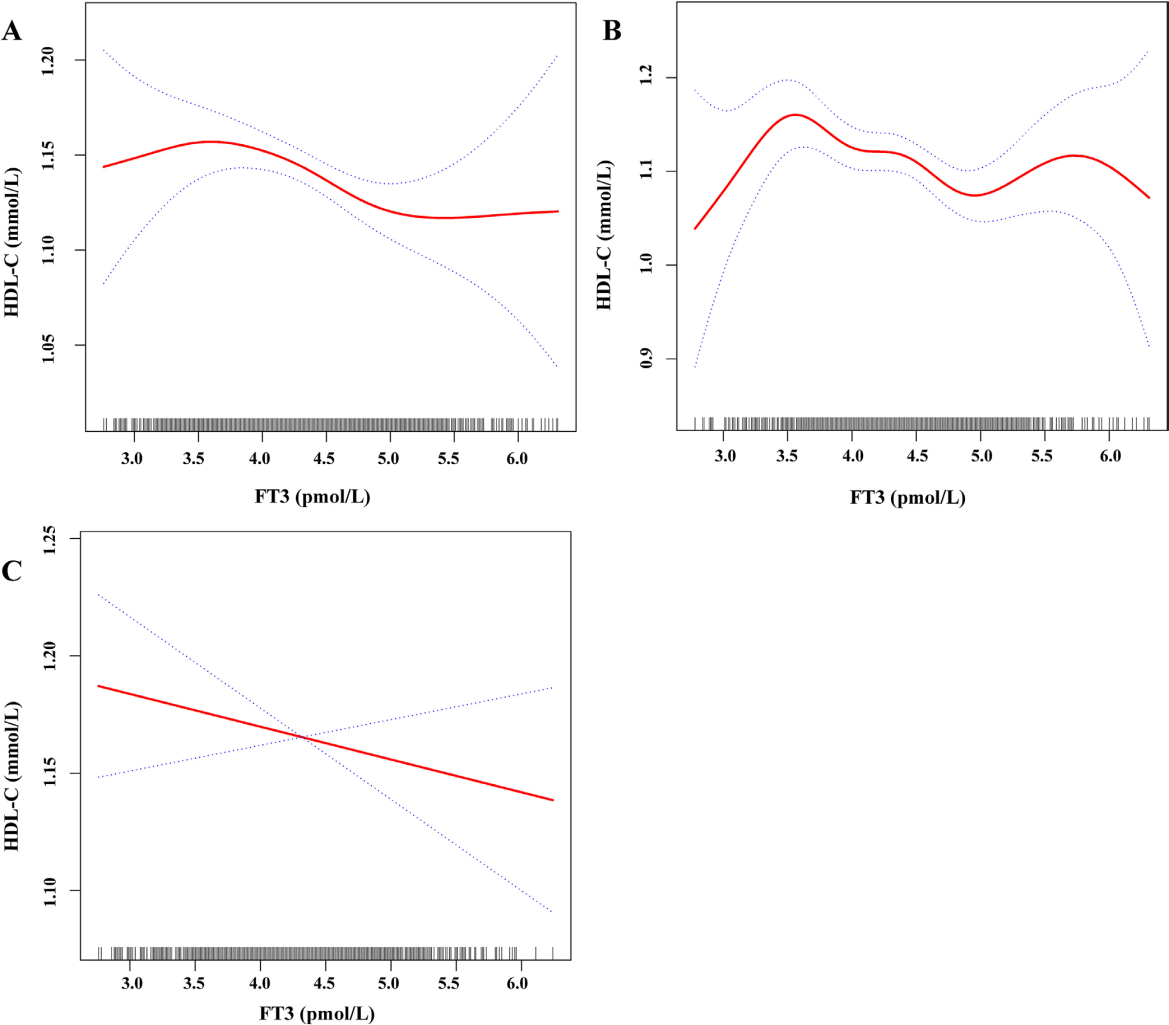
Smooth curve plot. (A) The result showed that the relationship between FT3 and HDL‐C was non‐linear, with the inflection point of FT3 being 3.48 pmol/L. (B) The result showed that the relationship between FT3 and HDL‐C was non‐linear in male subjects, with the first inflection point of FT3 being 3.51 pmol/L. (C) The result showed that the relationship between FT3 and HDL‐C in female subjects. (A) has been adjusted based on AGE; Sex; BMI; FT4; TSH; HbA1c. (B, C) have been adjusted based on AGE; BMI; FT4; TSH; HbA1c. The red curve is the actual fitted curve, and the upper and lower two blue dashed lines represent the 95% confidence interval.

Figure 3 shows the results of smooth curve fitting based on the generalized additive model. After adjusting for age, sex, BMI, FT4, TSH, and HbA1c, the smooth curve fitting between FT3 and HDL‐C showed a negative correlation and non‐linear relationship (Figure 3A), and HDL‐C tends to a stable state before the inflection point. After reaching the first inflection point, HDL‐C decreased with the increase of FT3 concentration (*p* = 0.044); HDL‐C continued to stabilize after the second inflection point, but the second inflection point was not significant (*p* = 0.106). The inflection point of the smooth curve is found by the maximum likelihood method. Based on the different trends before and after the inflection point, the subsequent segmentation analysis is carried out according to the inflection point FT3 = 3.48 pmol/L (Table 2).

**TABLE 2:** The result of the two‐piecewise linear regression model.

Inflection point of FT3	3.48		5.16	
	β‐coefficients (95% CI)^a^	*p*	β‐coefficients (95% CI)^a^	*p*
<Inflection point	0.11 (−0.02, 0.25)	0.0929	−0.04 (−0.06, −0.02)	0.0006
≥Inflection point	−0.03 (−0.05, −0.01)	0.0024	0.04 (−0.05, 0.13)	0.3650
P for log‐likelihood ratio test		0.044		0.106

*Note:* Effect: HDL‐C (mmol/L) Cause:FT3 (pmol/L).

Abbreviations: CI, confidence interval; FT3, for free triiodothyronine (pmol/L).

^a^ β‐Coefficients (95% CI) adjusted for age, sex, BMI, FT4, TSH, HbA1c.

Then we conducted a stratified analysis of gender. After adjusting for age, BMI, FT4, TSH, and HbA1c, the smooth curve fitting between FT3 and HDL‐C in male subjects also showed a non‐linear relationship (Figure 3B). HDL‐C increased with the increase of FT3 concentration before the first inflection point. After reaching the first inflection point, HDL‐C decreased with the increase of FT3 concentration. Based on the different trends before and after the inflection point, the subsequent segmentation analysis is carried out according to the inflection point FT3 = 3.51 pmol/L (Table 3). We did not observe a non‐linear relationship between FT3 and HDL‐C in female subjects (Figure 3C).

**TABLE 3:** The result of the two‐piecewise linear regression model in male subjects.

Inflection point of FT3	3.51	
	β‐coefficients (95% CI)^a^	*p*
<Inflection point	0.18 (−0.01, 0.37)	0.0668
≥Inflection point	−0.04 (−0.07, −0.01)	0.0027
P for log‐likelihood ratio test		0.031

*Note:* Effect: HDL‐C (mmol/L) Cause:FT3 (pmol/L).

Abbreviations: CI, confidence interval; FT3, for free triiodothyronine (pmol/L).

^a^ β‐Coefficients (95% CI) adjusted for age; BMI; FT4; TSH; HbA1c.

### 3.4 Logistic Regression Analyses of FT3 and HDL‐C

Table 4 shows the relationship between FT3 levels and HDL‐C in overall subjects. In the unadjusted and adjusted logistic regression model, FT3 was negatively correlated with HDL‐C. According to the inflection point FT3 = 3.48 pmol/L, in the unadjusted model, the β (95% CI) of the relationship between HDL‐C and total FT3 or FT3 levels in each subgroup were −0.03 (−0.05, −0.01; *p* = 0.0003); −0.01 (−0.05, 0.03; *p* = 0.6310). After adjusting for age, sex, and BMI, the β (95% CI) of the relationship between HDL‐C and total FT3 or FT3 levels in each subgroup were −0.02 (−0.04, −0.00; *p* = 0.023); 0.00 (−0.04, 0.04; *p* = 0.9353). After adjusting for age, sex, BMI, FT4, TSH, and HbA1c, the β (95% CI) of the relationship between HDL‐C and total FT3 or FT3 levels in each subgroup were −0.02 (−0.04, −0.00; *p* = 0.0162); −0.00 (−0.04, 0.04; *p* = 0.9527).

**TABLE 4:** Relationship between FT3 (pmol/L) and HDL‐C (mmol/L).

Outcome	Crude model		Model I		Model II	
	β (95% CI)	*p*	β (95% CI)	*p*	β (95% CI)	*p*
FT3	−0.03 (−0.05, −0.01)	0.0003	−0.02 (−0.04, −0.00)	0.023	−0.02 (−0.04, −0.00)	0.0162
FT3 (quartile)						
3.48	Reference		Reference		Reference	
3.48	−0.01 (−0.05, 0.03)	0.6310	0.00 (−0.04, 0.04)	0.9353	−0.00 (−0.04, 0.04)	0.9527
P for trend	0.6310		0.9353		0.9527	

*Note:* Model Iadjusted for age, sex, BMI. Model IIadjusted for age, sex, BMI, FT4, TSH and HbA1c.

Abbreviations: BMI, body mass index; CI, confidence interval; FT3, free triiodothyronine; FT4, free thyroxine; HbA1c, glycosylated hemoglobin; TSH, thyroid‐stimulating hormone.

Table 5 shows the relationship between FT3 levels and HDL‐C in male subjects. In the unadjusted and adjusted logistic regression model, FT3 was negatively correlated with HDL‐C. According to the inflection point FT3 = 3.51 pmol/L, in the unadjusted model, the β (95% CI) of the relationship between HDL‐C and total FT3 or FT3 levels in each subgroup was −0.04 (−0.06, −0.01; *p* = 0.0023); −0.01 (−0.08, 0.05; *p* = 0.6608). After adjusting for age and BMI, the β (95% CI) of the relationship between HDL‐C and total FT3 or FT3 levels in each subgroup was −0.03 (−0.05, −0.00; *p* = 0.0207); −0.01 (−0.07, 0.05; *p* = 0.715). After adjusting for Age, BMI, FT4, TSH, and HbA1c, the β (95% CI) of the relationship between HDL‐C and total FT3 or FT3 levels in each subgroup was −0.03 (−0.05, −0.00; *p* = 0.0199); −0.02 (−0.08, 0.04; *p* = 0.5688).

**TABLE 5:** Relationship between FT3 (pmol/L) and HDL‐C (mmol/l) in male subjects.

Outcome	Crude model		Model I		Model II	
	β (95% CI)	*p*	β (95% CI)	*p*	β (95% CI)	*p*
FT3	−0.04 (−0.06, −0.01)	0.0023	−0.03 (−0.05, −0.00)	0.0207	−0.03 (−0.05, −0.00)	0.0199
FT3 (quartile)						
3.51	Reference		Reference		Reference	
3.51	−0.01 (−0.08, 0.05)	0.6608	−0.01 (−0.07, 0.05)	0.7150	−0.02 (−0.08, 0.04)	0.5688
P for trend	0.6608		0.7150		0.5688	

*Note:* Model I adjusted for age, BMI. Model II adjusted for age, BMI, FT4, TSH, and HbA1c.

Abbreviations: BMI, body mass index; CI, confidence interval; FT3, free triiodothyronine; FT4, free thyroxine; HbA1c, glycosylated hemoglobin; TSH, thyroid‐stimulating hormone.

## 4 Discussion

Thyroid dysfunction is a risk factor for cardiovascular disease, and thyroid hormone (TH) has multiple effects on lipid synthesis, mobilization, and degradation, indicating that TH affects the development of dyslipidemia. TH promotes fat synthesis and accelerates fat consumption, but decomposition is greater than synthesis. Therefore, when hyperthyroidism occurs, TC, LDL‐C, and TG decrease. Conversely, when hypothyroidism occurs, both fat synthesis and decomposition decrease, but the degree of degradation is greater, ultimately leading to an increase in TC, LDL‐C, and TG. FT3 can induce the synthesis of various fat metabolism enzymes. When TH is deficient, one of the reasons for the increase in medium‐and low‐density lipoprotein cholesterol is the decrease in low‐density lipoprotein receptors and cholesterol clearance rate. At the same time, the activity of liver lipase decreases, the conversion of medium‐density lipoprotein to low‐density lipoprotein slows down, and the clearance rate of triglycerides decreases [18, 19, 20], but most subjects at risk of cardiovascular disease have normal thyroid function in clinical settings. Recently, the relationship between thyroid hormone and atherosclerosis in people with normal thyroid function has attracted wide attention.

To minimise selection bias, data from all patients diagnosed with T2DM in the Endocrinology Department of the Fourth Affiliated Hospital of Harbin Medical University between June 2022 and October 2023 were collected non‐selectively and consecutively. A total of 4752 patients were initially included. After applying exclusion criteria, 3011 patients were retained for analysis (average age 56.92 ± 12.56 years), including 1430 males (average age 53.91 ± 12.78 years) and 1581 females (average age 59.65 ± 11.72 years). Of the 1430 male T2DM patients, 526 (36.78%) patients' HDL‐C ≤ 1.0 mmol/L. Compared with the male Low HDL‐C group, the High HDL‐C group had lower levels of HbA1c and FBG (*p* < 0.01). There were significant differences in the levels of Age, BMI, FT3, FT4, sCr, AST, ALT, TC, TG, and LDL‐C between the two groups (*p* < 0.05). Among 1581 female T2DM patients, 1181 (74.70%) patients' HDL‐C ≤ 1.3 mmol/L. There were significant differences in Age, BMI, SBP, sCr, TC, TG, and LDL‐C levels between the two groups (*p* < 0.05). Variables with non‐zero coefficients in the LASSO regression model are considered to be related to HDL‐C. They were selected for further studies, including age, sex, BMI, FT3, FT4, TSH, and HbA1c. Meanwhile, as there is no difference in FT3 between the male and female groups, our analysis of the correlation between FT3 and HDL‐C will be more objective. Therefore, we ultimately chose FT3 as the observation indicator. In the subsequent statistical analysis, we will adjust for age, gender, BMI, FT4, TSH, and HbA1c before conducting the analysis.

Giandalia et al. [9] believe that TH is associated with visceral obesity and higher triglyceride concentrations in T2DM patients. The research results of Triolo et al. [11] indicate that in chronic hyperglycemia, higher TH can lead to increased plasma cholesterol ester transport. All these indicate that relatively low but clinically normal thyroid function may also affect the blood lipid status and atherosclerosis susceptibility of T2DM patients. Our research results are basically consistent with the above research results. The results of this study show that among T2DM patients with normal thyroid function (serum FT3, FT4, TSH were within the normal range, and the reference value of normal range of FT3 in the author's hospital was 2.76–6.45 pmol/L, FT4 was 11.20–23.81 pmol/L, TSH was 0.35–5.10 mIU/L), after adjusting for age, sex, BMI, FT4, TSH, and HbA1c, there is a negative correlation and non‐linear relationship between FT3 and HDL‐C. When FT3 is < 3.48 pmol/L, HDL‐C tends to a stable state; when FT3 is ≥ 3.48 pmol/L, HDL‐C decreases with the increase of FT3 concentration. This indicates that in T2DM patients with normal thyroid function, the effect of FT3 on HDL‐C is in a dynamic balance of bidirectional regulation. When FT3 ≥ 3.48 pmol/L, the basal metabolic rate relatively increases, and TH promotes HDL‐C decomposition ability gradually greater than synthesis ability, resulting in a gradual decrease in HDL‐C. Extending to the pathological state, when TH replacement therapy is needed, controlling hormones within a reasonable range is beneficial for maintaining a dynamic balance of HDL‐C metabolism; for example, controlling FT3 within the range of < 3.48 pmol/L is optimal. We have come to the conclusion that FT3 within the range of 2.76 to 3.48 pmol/L may be most beneficial for mitigating the progression of cardiovascular disease in patients with T2DM. Therefore, doctors can purposefully control FT3 within this range to delay the progress of cardiovascular disease in type 2 diabetes. After understanding this range, patients can seek medical attention promptly to control FT3 within this range.

FT3 and HDL‐C are negatively correlated; one of the reasons may be that both are the result of autoimmune activation involving lipoprotein (a), and then the level of lipoprotein (a) is “reduced”, which is the determinant of new diabetes, accompanied by low circulating TC and HDL‐C, as well as autoimmune complexes involving thyroid hormones. In patients with T2DM and normal thyroid function, the above variables may tend to normalize due to weakened autoimmune processes and decreased levels of lipoprotein (a) [21, 22, 23].

In addition, we will extend these results to gender analysis in more detail. We evaluated the basic information of T2DM patients in the adult population. According to the “Guidelines for the Prevention and Treatment of Type 2 Diabetes in China” [13], HDL‐C should be controlled at > 1.0 mmol/L in male patients with T2DM; and should be controlled at > 1.3 mmol/L in female patients with T2DM. We found that after adjusting for the effects of age, BMI, FT4, TSH, and HbA1c, there was a negative correlation and non‐linear relationship between FT3 and HDL‐C in male subjects. When FT3 < 3.51 pmol/L, HDL‐C increased with the increase of FT3 concentration; When FT3 ≥ 3.51 pmol/L, HDL‐C decreased with the increase of FT3 concentration. We did not observe a non‐linear relationship between FT3 and HDL‐C in female subjects. This may be due to the fact that the sample size of High HDL‐C group and Low HDL‐C group is similar in male subjects, while there is a large difference in sample size between High HDL‐C group and Low HDL‐C group in female subjects. Subsequently, we will further expand the sample size to clarify a more objective range of FT3.

There are some limitations in our research. First of all, as this study is a cross‐sectional study, it is not possible to infer a causal relationship from it. the participants in this study were all hospitalized patients, which may not fully represent the correlation between FT3 and HDL‐C in outpatient T2DM patients. Moreover, since the data was collected during hospitalization, the results of indicators such as HDL‐C and FT3 were only tested once, which may result in a certain degree of error. Finally, due to limitations in the sample size of the study, the results may need further improvement. However, current research on the relationship between FT3 and HDL‐C is still limited, and our research results are only preliminary explorations. In future research, we will further expand the sample size and conduct multicenter studies to improve the results of this study.

## Author Contributions

J.X. and J.H. conceived and designed the study, and were involved in data acquisition and analysis. Data interpretation was done by S.Z. Critical revision of important intellectual content was carried out by Y.W. All the authors have read the final manuscript and approved it for publication.

## Ethics Statement

The study was approved by the Ethics Committee of the fourth affiliated Hospital of Harbin Medical University and was conducted in accordance with the ethical standards of institutions and national research committees, as well as the 1964 Helsinki Declaration and its subsequent amendments or similar ethical standards. Ethical approval no. 2023‐Ethical Review‐26.

## Consent

As the study involved the retrospective analysis of clinical data, the requirement for written informed consent was waived.

## Conflicts of Interest

The authors declare no conflicts of interest.

## Data Availability

The data sets generated during and/or analyzed during the current study are available from the corresponding author upon reasonable request.

## References

[1] T. Ichiki , “Thyroid Hormone and Atherosclerosis,” Vascular Pharmacology 52 (2010): 151–156. 19808101 10.1016/j.vph.2009.09.004

[2] M. Murolo , O. Di Vincenzo , A. G. Cicatiello , et al., “Cardiovascular and Neuronal Consequences of Thyroid Hormones Alterations in the Ischemic Stroke,” Metabolites 13, no. 1 (2022): 22. 36676947 10.3390/metabo13010022 PMC9863748

[3] X. Wang , Z. Wu , Y. Liu , et al., “The Role of Thyroid‐Stimulating Hormone in Regulating Lipid Metabolism: Implications for Body‐Brain Communication,” Neurobiology of Disease 201 (2024): 106658, 10.1016/j.nbd.2024.106658. 39236910

[4] J. Garduño‐Garcia Jde , U. Alvirde‐Garcia , G. López‐Carrasco , et al., “TSH and Free Thyroxine Concentrations Are Associated With Differing Metabolic Markers in Euthyroid Subjects,” European Journal of Endocrinology 163 (2010): 273–278. 20516204 10.1530/EJE-10-0312

[5] J. Liu , Q. Fu , R. Su , et al., “Association Between Nontraditional Lipid Parameters and the Risk of Type 2 Diabetes and Prediabetes in Patients With Nonalcoholic Fatty Liver Disease: From the National Health and Nutrition Examination Survey 2017–2020,” Frontiers in Endocrinology 15 (2024): 1460280. 39280011 10.3389/fendo.2024.1460280 PMC11392789

[6] T. Salmen , V. A. Pietrosel , D. Reurean‐Pintilei , et al., “Assessing Cardiovascular Target Attainment in Type 2 Diabetes Mellitus Patients in Tertiary Diabetes Center in Romania,” Pharmaceuticals (Basel) 17, no. 9 (2024): 1249. 39338411 10.3390/ph17091249 PMC11434711

[7] R. Kadiyala , R. Peter , and O. E. Okosieme , “Thyroid Dysfunction in Patients With Diabetes: Clinical Implications and Screening Strategies,” International Journal of Clinical Practice 64 (2010): 1130–1139. 20642711 10.1111/j.1742-1241.2010.02376.x

[8] S. A. Chubb , W. A. Davis , and T. M. Davis , “Interactions Among Thyroid Function, Insulin Sensitivity, and Serum Lipid Concentrations: The Fremantle Diabetes Study,” Journal of Clinical Endocrinology and Metabolism 90 (2005): 5317–5320. 15985488 10.1210/jc.2005-0298

[9] A. Giandalia , G. T. Russo , E. L. Romeo , et al., “Influence of High‐Normal Serum TSH Levels on Major Cardiovascular Risk Factors and Visceral Adiposity Index in Euthyroid Type 2 Diabetic Subjects,” Endocrine 47, no. 1 (2014): 152–160, 10.1007/s12020-013-0137-2. 24385267

[10] J. Du , X. Zhao , X. Xu , et al., “Association Between Thyroid Parameters and Subclinical Atherosclerosis in Hospitalised Euthyroid Patients With Type 2 Diabetes Mellitus,” Diabetes, Metabolic Syndrome and Obesity 16 (2023): 3163–3171. 10.2147/DMSO.S429941 PMC10578159 37849978

[11] M. Triolo , A. J. Kwakernaak , F. G. Perton , R. de Vries , G. M. Dallinga‐Thie , and R. P. F. Dullaart , “Low Normal Thyroid Function Enhances Plasma Cholesteryl Ester Transfer in Type 2 Diabetes Mellitus,” Atherosclerosis 228 (2013): 466–471. 23591416 10.1016/j.atherosclerosis.2013.03.009

[12] K. G. Alberti and P. Z. Zimmet , “Definition, Diagnosis and Classification of Diabetes Mellitus and Its Complications. Part 1: Diagnosis and Classification of Diabetes Mellitus Provisional Report of a WHO Consultation,” Diabetic Medicine 15, no. 7 (1998): 539–553. 9686693 10.1002/(SICI)1096-9136(199807)15:7<539::AID-DIA668>3.0.CO;2-S

[13] Microvascular Complications Group of Diabetes Credit Association of Chinese Medical Association , “Guidelines for the Prevention and Treatment of Diabetes and Kidney Disease in China,” Chinese Journal of Diabetes Mellitus 13 (2021): 762–784.

[14] L. Marino , A. Kim , B. Ni , and F. S. Celi , “Thyroid Hormone Action and Liver Disease, a Complex Interplay,” Hepatology 81, no. 2 (2025): 651–669. 37535802 10.1097/HEP.0000000000000551 PMC11737129

[15] J. Friedman , T. Hastie , and R. Tibshirani , “Regularization Paths for Generalized Linear Models via Coordinate Descent,” Journal of Statistical Software 33, no. 1 (2010): 1–22. 20808728 PMC2929880

[16] W. Sauerbrei , P. Royston , and H. Binder , “Selection of Important Variables and Determination of Functional Form for Continuous Predictors in Multivariable Model Building,” Statistics in Medicine 26, no. 30 (2007): 5512–5528. 18058845 10.1002/sim.3148

[17] A. C. Kidd , M. McGettrick , S. Tsim , D. L. Halligan , M. Bylesjo , and K. G. Blyth , “Survival Prediction in Mesothelioma Using a Scalable Lasso Regression Model: Instructions for Use and Initial Performance Using Clinical Predictors,” BMJ Open Respiratory Research 5, no. 1 (2018): e000240. 10.1136/bmjresp-2017-000240 PMC5812388 29468073

[18] R. He , H. Bi , J. He , et al., “Thyroid Hormones and Oxidative Stress Moderated the Association Between Urinary Phthalate Metabolites and Cardiovascular Risk Factors,” Environmental Pollution 362 (2024): 124927. 39265773 10.1016/j.envpol.2024.124927

[19] M. Sommer‐Ballarini , T. H. Nguyen , L. Pletsch‐Borba , et al., “Impact of Peripheral Thyroid Hormone Balance on Liver Fat: Insights From the NutriAct Trial,” European Journal of Endocrinology 191, no. 2 (2024): 183–191. 39049801 10.1093/ejendo/lvae093

[20] X. Li , X. He , X. Lin , et al., “Effects of Bisphenols on Lipid Metabolism and Neuro‐ Cardiovascular Toxicity in Marine Medaka Larvae,” Aquatic Toxicology 259 (2023): 106551. 37156703 10.1016/j.aquatox.2023.106551

[21] A. Onat , G. Can , S. Murat , G. Ciçek , E. Örnek , and H. Yüksel , “Aggregation of Lipoprotein(a) to Apolipoprotein A‐I Underlying HDL Dysfunction as a Major Coronary Risk Factor,” Anadolu Kardiyoloji Dergisi 13 (2013): 543–551. 23835300 10.5152/akd.2013.175

[22] Q. Zhu , Y. Zheng , X. Lang , et al., “Prevalence and Correlates of Dyslipidemia in First‐Episode and Drug‐Naïve Major Depressive Disorder Patients With Comorbid Abnormal Glucose Metabolism: Sex Differences,” Frontiers in Psychiatry 14 (2023): 1101865. 36793942 10.3389/fpsyt.2023.1101865 PMC9922762

[23] H. Chen , J. Wu , and R. Lyu , “Expressions of Glycemic Parameters, Lipid Profile, and Thyroid Hormone in Patients With Type 2 Diabetes Mellitus and Their Correlation,” Immunity, Inflammation and Disease 12, no. 7 (2024): e1282. 38967365 10.1002/iid3.1282 PMC11225078

